# A promising azeotrope-like mosquito repellent blend

**DOI:** 10.1038/s41598-017-10548-y

**Published:** 2017-08-31

**Authors:** Homa Izadi, Walter W Focke, Erfan Asaadi, Rajendra Maharaj, Jannie Pretorius, Mattheüs Theodor Loots

**Affiliations:** 1 Institute of Applied Materials, Department of Chemical Engineering, University of Pretoria, Pretoria, South Africa; 2 Institute for Sustainable Malaria Control, University of Pretoria, Pretoria, South Africa; 3 Office of Malaria Research, Medical Research Council, Durban, South Africa; 4 Department of Chemistry and Supercomputer Centre, University of Pretoria, Pretoria, South Africa; 5 Department of Statistics, University of Pretoria, Pretoria, South Africa

## Abstract

Topical repellents play a key role in reducing the outdoor transmission of mosquito-borne diseases by reducing human-vector contact. Excellent repellents are available, but there is always room for improvement. This article reports on a particularly effective binary repellent blend of ethyl butylacetylaminopropionate and nonanoic acid. A composition containing 25 mol% of the acid exhibits negative pseudo-azeotrope behaviour at 50 °C, meaning that the liquid vapour pressure is lower than that of the parent compounds and evaporation occurs without a change in the liquid composition. In tests performed using the South African Medical Research Council’s cup-on-arm procedure, this mixture provided better protection for a longer time than the “gold standard of mosquito repellents”, namely N,N-diethyl-m-toluamide, commonly known as DEET.

## Introduction

Mosquitoes are insect vectors of deadly diseases such as malaria, dengue fever, Zika virus, lymphatic filariasis and West Nile fever. They infect hundreds of millions of people, causing immense suffering and up to a million deaths per annum^1^. These disease transmissions can be reduced by using, for example, topical mosquito repellents that prevent or at least reduce contact between the blood-seeking female mosquitoes and humans^2^.

N,N-diethyl-m-toluamide (DEET) is considered the gold standard to which all other mosquito repellents are compared^3^. However, emergent resistance and negative consumer perceptions related to aspects such as odour, high adsorption rate, oily feel on the skin and even skin irritation^3, 4^ indicate a growing need for replacements. Commercialising new active compounds and establishing their safety for human use are time-consuming and expensive activities^5^. The discovery of synergistic mixtures of approved repellents could provide a faster route to public use. In this context, the present article reports on the discovery of a special blend of two repellents that exhibits pseudo-azeotrope behaviour with improved repellent efficacy and persistence.

The repellents, in commercial use, tend to be low-volatility liquids. They are usually applied directly to the skin from where they slowly evaporate into the ambient air. The released vapours are swept away by convection currents caused by air drafts and limb motions. For fixed convection conditions, theoretical considerations predict that (and experimental observations confirm this), for practical purposes, the rate of mass loss varies linearly with the vapour pressure of the evaporating liquid^6^. This implies that strategies that effectively reduce the apparent vapour pressure of the repellent could help to produce a longer-lasting effect. The caveat is that suppression of the volatility should not proceed to the point where the amount of repellent present is insufficient to provide adequate protection. An elegant way to give effect to this idea is to blend the repellent with another suitable compound with which it forms a negative pseudo-azeotrope. This concept is explained in Supplementary Information (SI. 1).

## Azeotrope phenomena

Azeotrope phenomena are well known in phase equilibrium thermodynamics, but less so in the field of repellent applications. Therefore, a brief overview is in order. More detailed discussions can be found elsewhere^7, 8^. A conventional azeotrope is a mixture of two or more substances that retains the same composition in the vapour state as in the liquid state when distilled or partially evaporated at a fixed temperature or pressure^8^. At isobaric conditions, the presence of an azeotrope is manifested by an extreme value in the temperature-composition curve. Higher boiling and lower boiling (positive) azeotropes feature boiling points that lie above and below the boiling points of the pure components respectively.

Azeotropes pose limits on equilibrium separation processes relying on distillation because the vapour and liquid compositions are identical. However, this constant composition effect can be an advantage for products that rely on evaporation to achieve a desired effect. For example, a fragrance or perfume formulation that acts like an azeotrope could conceivably maintain the same odour for extended periods of time. In reality, real processes are rarely entirely equilibrium controlled^8^. True equilibrium is, at best, approached only at the air-liquid-interface and not in the whole system^9^. Nevertheless, in practical repellent evaporation situations, the gradients that develop in the intensive variables may set up a steady-state situation such that the vapour that is released has the same composition as the evaporating liquid, i.e. a “pseudo-azeotrope” is formed^8^. Obviously, this depends on the evaporation conditions and relevant transport properties.

The essential requirement for the formation of a negative azeotrope is that there should be sufficiently strong attractive interactions between the unlike molecules. Based on this insight, it was discovered that ethyl butylacetylaminopropionate, commercially known as IR3535, in combination with nonanoic acid satisfies this hypothesis. The justification for selecting this particular pair will be explained later. Interestingly, it transpired that this binary mixture features double pseudo-azeotropy, i.e. there are actually two different pseudo-azeotrope compositions. This is very unusual as few double-azeotrope systems are known for binary mixtures^10^.

Consider the evaporation, into air, of a binary liquid mixture containing two components, A and B. It is assumed that the air in direct contact with the liquid is in equilibrium with the liquid but that this vapour is continuously removed by air convection currents. Let the mole fractions of component A in the vapour phase (without the air components) and in the liquid phase be denoted by *y*_*A*_ and *x*_*A*_ respectively.

**Figure 1:**
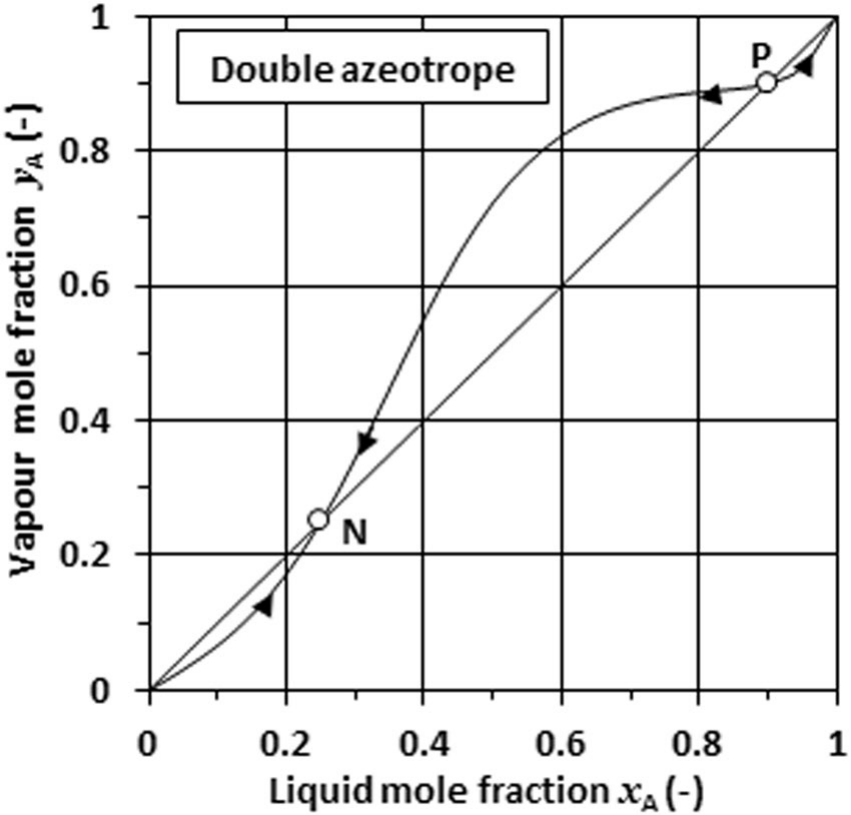
Typical *y*_A_ *vs*. *x*_A_ phase diagrams for a double azeotrope. Point N shows the negative azeotrope and point P shows the position of the positive azeotrope.

Figure 1 shows a *y*_A_ vs. *x*_A_ phase diagram for a binary system which features a double azeotrope, i.e. a positive and a negative one. The azeotrope compositions correspond to the points where the equilibrium curve crosses the *y*_A_ = *x*_A_ line. Consider the evaporation of a liquid mixture with a composition that exceeds the positive azeotrope. Figure 1 (point P) shows that the composition of the released vapour contains less of component A than is present in the liquid. Therefore, as evaporation proceeds, the liquid will become enriched with respect to component A. In other words, the liquid composition will change towards the pure component A as the liquid is progressively depleted. Next consider the evaporation of a mixture with a composition just lower than the positive azeotrope composition. Now the equilibrium curve lies above the *y*_A_ = *x*_A_ line in Fig. 1. This implies that the released vapour is enriched in component A with respect to the liquid composition. So, over time as the liquid evaporates, the composition of the liquid will drift towards pure component B. The implication is that this positive pseudo-azeotrope composition represents an unstable composition node.

The opposite is true for the negative pseudo-azeotrope system defined by point N in Fig. 1. A similar analysis leads to the conclusion that, in this case, the azeotrope composition is an attractor. All liquid mixtures will over time, as the liquid evaporates, tend towards the negative pseudo-azeotrope composition. A special feature of the negative pseudo-azeotrope blend composition is that the relative composition of the components in the evaporating vapour corresponds to that of the liquid. In other words, the composition of the remaining liquid will not change as it evaporates over time. In summary, the negative pseudo-azeotrope repellent composition has two desirable features: (i) it represents the mixture composition that exhibits the lowest vapour pressure at the given temperature, and (ii) it behaves just as if it is a pure compound, i.e. the composition stays constant during evaporation.

## Results and Discussion

**Figure 2:**
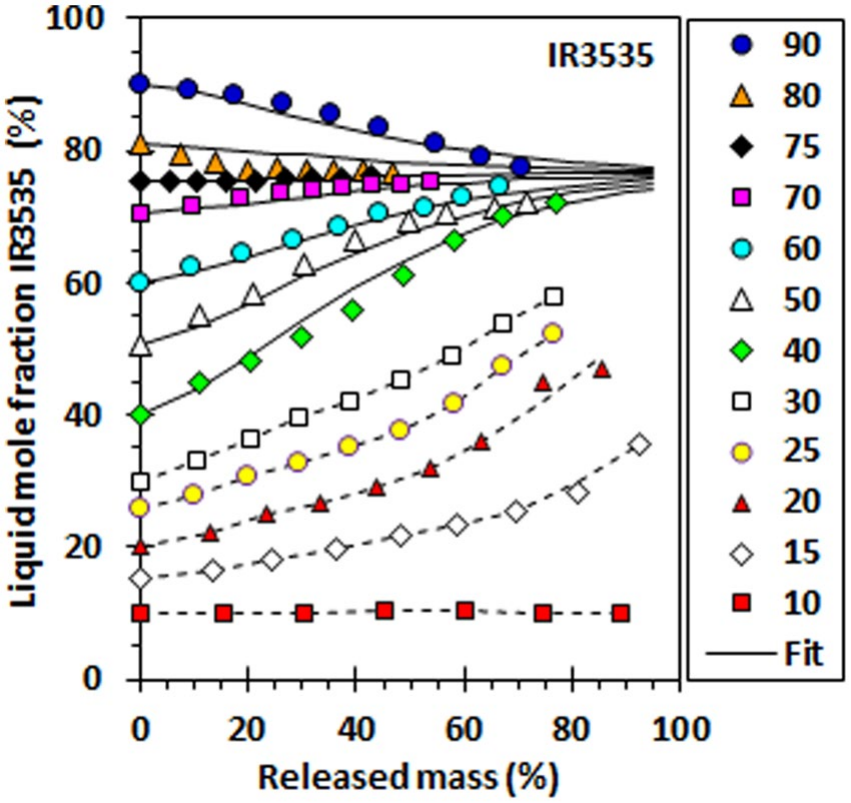
The change in the composition of the liquid phase vs. the released mass as evaporation proceeds. The key indicates the initial IR3535 content of the mixtures. The solid lines represent non-linear regression fits of an Avrami-like equation to the data with an initial IR3535 content of 40 mol% or higher. See Supplementary Information (SI. 10) for details. The Bootstrap estimate for the azeotrope composition is 76.7 mol% and the 95% confidence interval is (75.9, 77.5).

Ethyl butylacetylaminopropionate (IR3535) features an ester and a tertiary amide functional group, both of which can act as hydrogen bond acceptors. Carboxylic acids are capable of forming strong interactions with such groups^11^. Figure 2 summarises the data collected for mixtures of nonanoic acid and ethyl butylacetylaminopropionate as they evaporated from shallow dishes placed in a convection oven set at 50 °C. The change with time in the liquid composition was followed using Fourier transform infrared spectroscopy (FTIR). Figure 2 shows how the liquid composition changed as a function of the liquid fraction that remained. The composition of the mixture containing 10 mol% IR3535 remained approximately constant as the mixture evaporated. However, the compositions of the other mixtures tested converged towards a common concentration, approximately 77 mol% IR3535. As discussed above, this kind of drift towards a unique composition is expected for a mixture that forms a negative azeotrope^12^.

**Figure 3:**
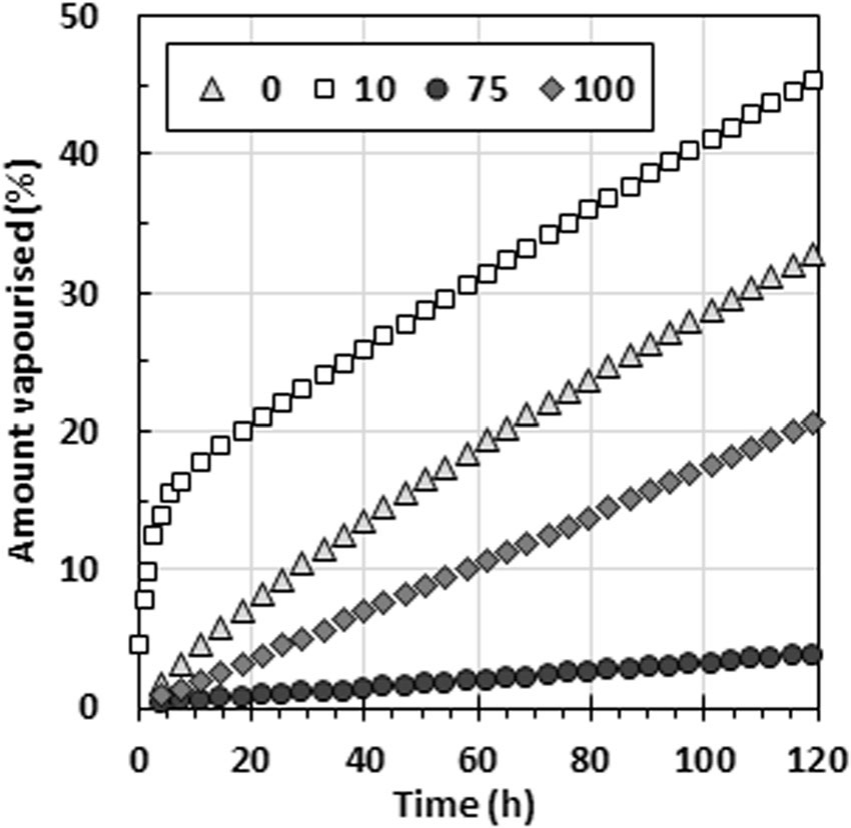
Isothermal evaporation of ethyl butylacetylaminopropionate and nonanoic acid mixtures at 50 °C.

Figure 3 shows isothermal evaporation results measured using open pans in a thermogravimetric analyser (TGA). It compares the behaviour of two binary mixtures of ethyl butylacetylaminopropionate and nonanoic acid with the response observed for the neat compounds. The mixture containing 77 mol% IR3535 evaporated significantly slower than the pure compounds, while the sample that contained close to 10 mol% evaporated significantly faster. Relatively speaking, initially the latter showed a very fast mass loss, but after about 12 h the mass loss rate (indicated by the slope of the curve) stabilised at a value similar to that of the neat nonanoic acid. The implication is that the composition of the remaining liquid for this mixture approached a composition close to that of the neat acid.

The data presented in Figs 2 and 3 support the notion that the ethyl butylacetylaminopropionate and nonanoic acid binary system features two pseudo-azeotrope compositions at the temperature of 50 °C. They are called pseudo-azeotropes as the evaporation occurs into the air under dynamic, rather than equilibrium, conditions. Note that the released vapour at every instant of time is, in effect, rapidly removed by a combination of diffusion and convection mechanisms.

**Figure 4:**
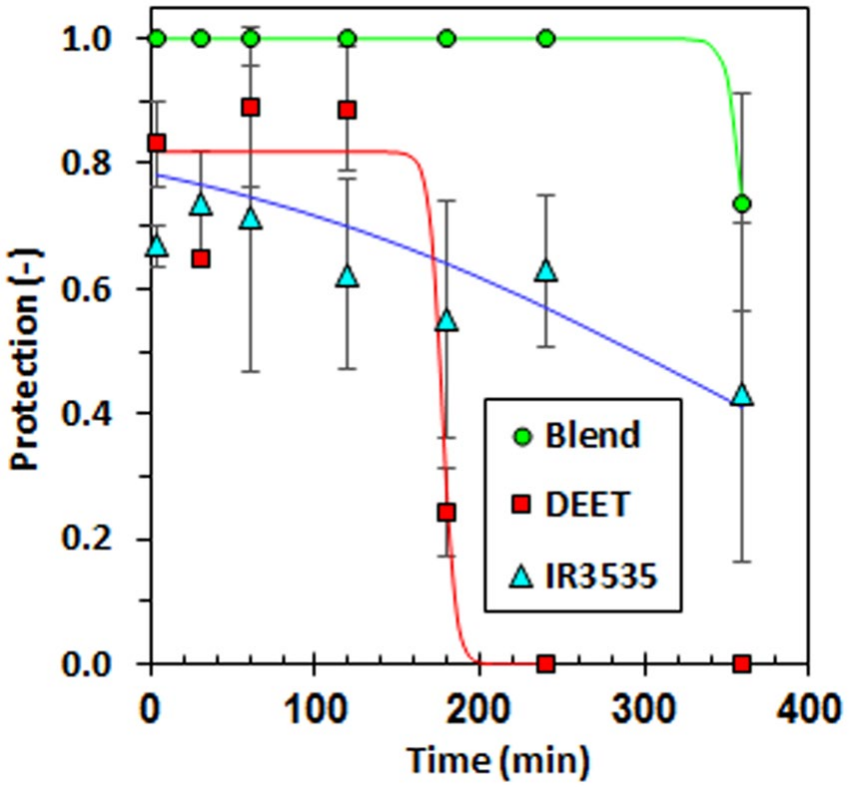
Temporal protection of the negative pseudo-azeotrope, IR3535 and DEET tested after application on the skin. The solid lines represent non-linear regression fits of sigmoidal curves to the data. See Supplementary Information (SI. 10) for details.

Suppressing the volatility of the repellent has the potential to increase the protection time. However, if it is reduced too much, there is a risk that the quantities released will be insufficient to provide effective repellence. Figure 4 compares the performance of the negative pseudo-azeotrope blend and the neat repellents DEET and IR3535 in a standard topical repellence test. During the first two hours of exposure, DEET and IR3535 showed a relatively high level of protection; however, after the third hour, the protection provided by DEET showed a sudden drop, whereas the protection provided by IR3535 showed a more gradual decrease. DEET failed to show any protection four hours after application, but IR3535 was still 62% effective relative to the negative control. In comparison to these two repellents, the IR3535-nonanoic acid negative pseudo-azeotrope (i.e. the mixture containing 75 mol% IR3535) showed superior repellence and a longer-lasting effect. It provided essentially full protection for up to four hours.

The application of IR3535 and DEET simply repelled the mosquitoes. In contrast, the pseudo-azeotropic also showed a knock-down effect on the test mosquitoes, this even leading to mortality. The latter could be important for two reasons. First, killing the repelled mosquitoes should help to prevent the development of resistance against active ingredients over time. Secondly, preventing outdoor transmission of malaria infection cannot rely solely on preventing protected people from being bitten as the mosquitoes will simply move on to other human targets. It is, therefore, desirable that the repellence is combined with a killing effect. Binary mixtures that feature two different azeotrope compositions are quite rare^10^. FTIR spectroscopy and molecular modelling studies were therefore conducted to gain an understanding of this unusual behaviour.

## Vibrational analysis

**Figure 5:**
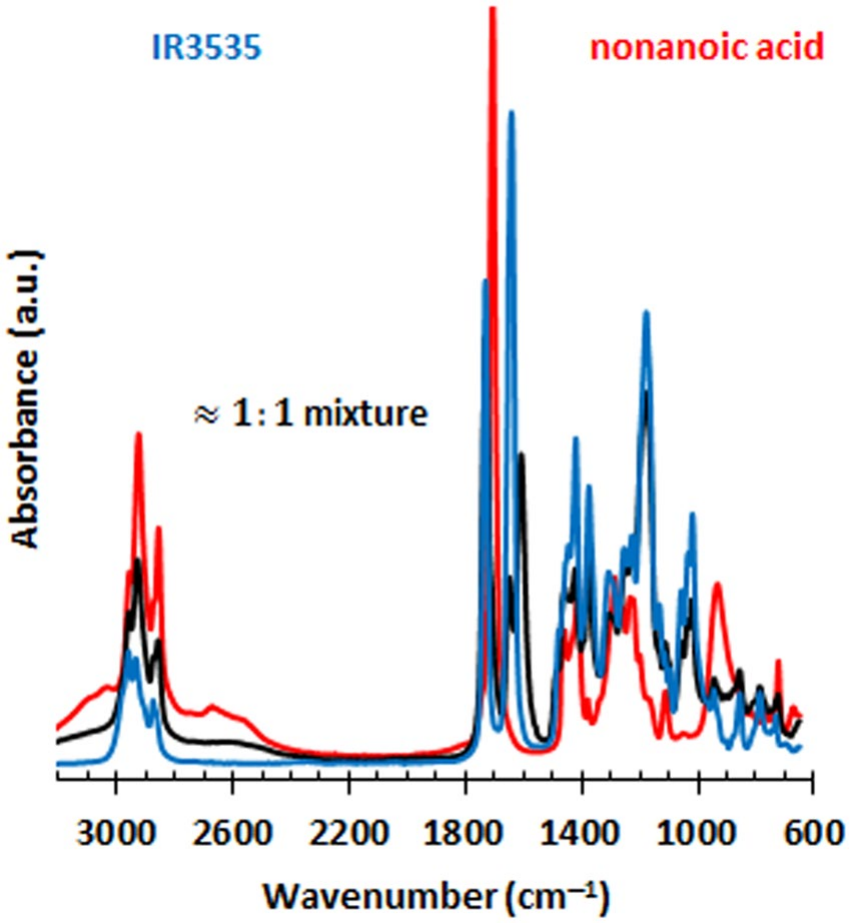
FTIR spectra for neat nonanoic acid (in red), IR3535 (in blue) and a near-equimolar mixture (in black).

The FTIR spectra recorded for nonanoic acid, IR3535 and a near-equimolar liquid mixture are presented in Fig. 5. For the present discussion, the most important peaks in the spectrum for IR3535 are the two carbonyl stretch vibrations associated with the ester (1735 cm^−1^) and amide (1638 cm^−1^) functional groups respectively^13, 14^. For a detailed description of the spectral features see Supplementary Information (SI. 2).

**Figure 6:**
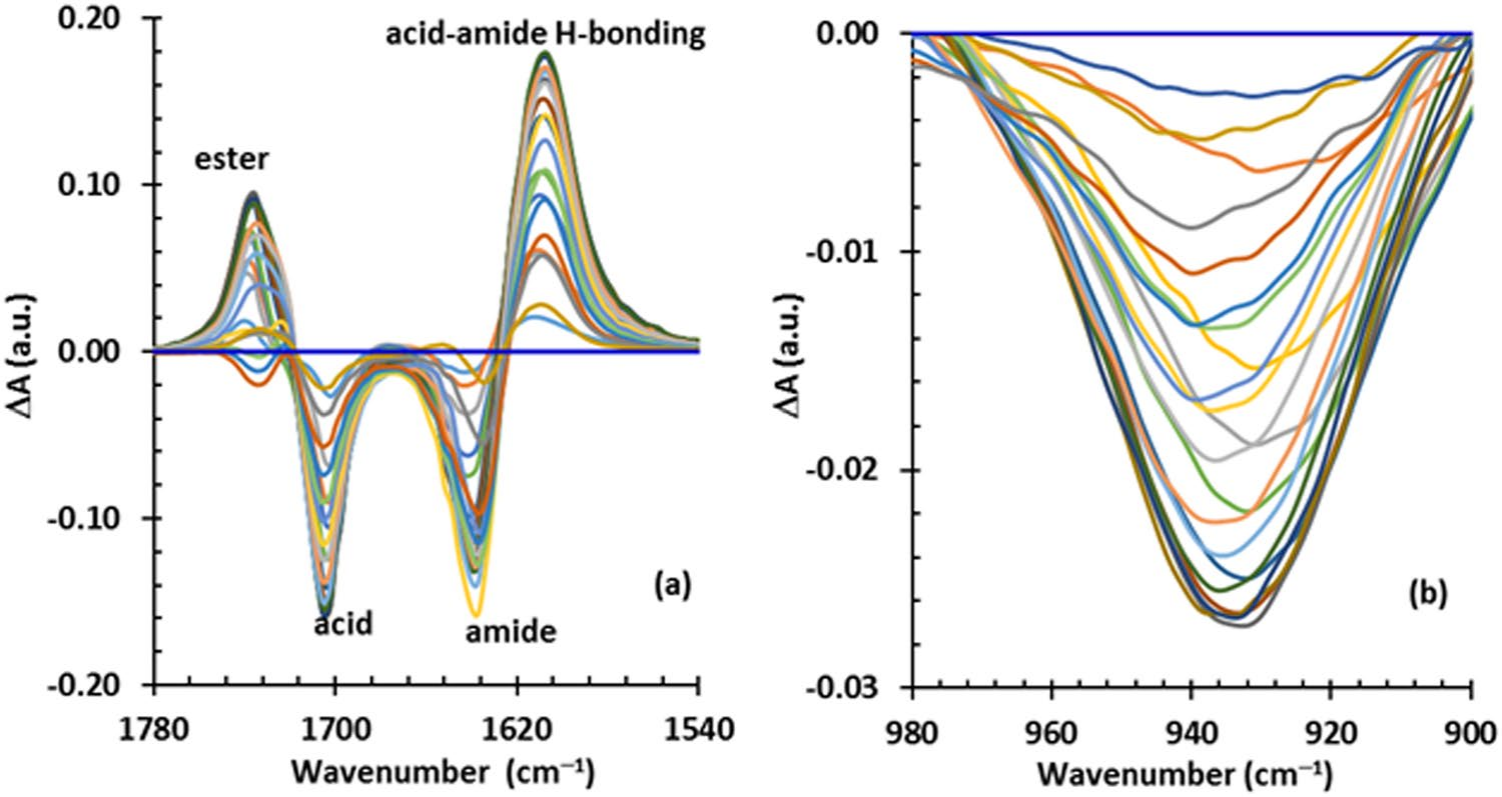
The variation of the absorbance mixing function (∆A) with composition. (**a**) Carbonyl stretch region showing ester, acid and amide C=O in addition to C=O involved in intermolecular acid-amide hydrogen bonding, and (**b**) “dimer acid” band.

If there were no intermolecular interactions, the FTIR spectra of mixtures should approximate mole fraction-weighted combinations of the parent compound spectra. Data interpretation may be assisted by plotting the spectral residuals, i.e. the difference between the spectrum for the actual mixture and such a combination of the pure components’ spectra^15^. The spectral residuals in a selected range of wavenumbers for a set of IR3535-nonanoic acid binary mixtures were calculated (see Supplementary Information (SI. 3) and are presented in Fig. 6. The negative values for Δ*A*_*mix*_ for the acid (1705 cm^−1^) and amide (1638 cm^−1^) carbonyl stretch vibrations indicate that these two functional groups changed because they interacted. In this case, they actually engage in a hydrogen bonding interaction that gives rise to a new absorption peak, which is reflected in the positive deviations in Δ*A*_*mix*_ at 1608 cm^−1^ in Fig. 6a. Interestingly, the ester carbonyl band (1735 cm^−1^) also shows positive deviations in Δ*A*_*mix*_ at lower IR3535 concentrations. Beyond that, it also shows a shift of the peak positions to higher wavenumbers, consistent with a decrease in the intermolecular interactions with other functional groups.

**Figure 7:**
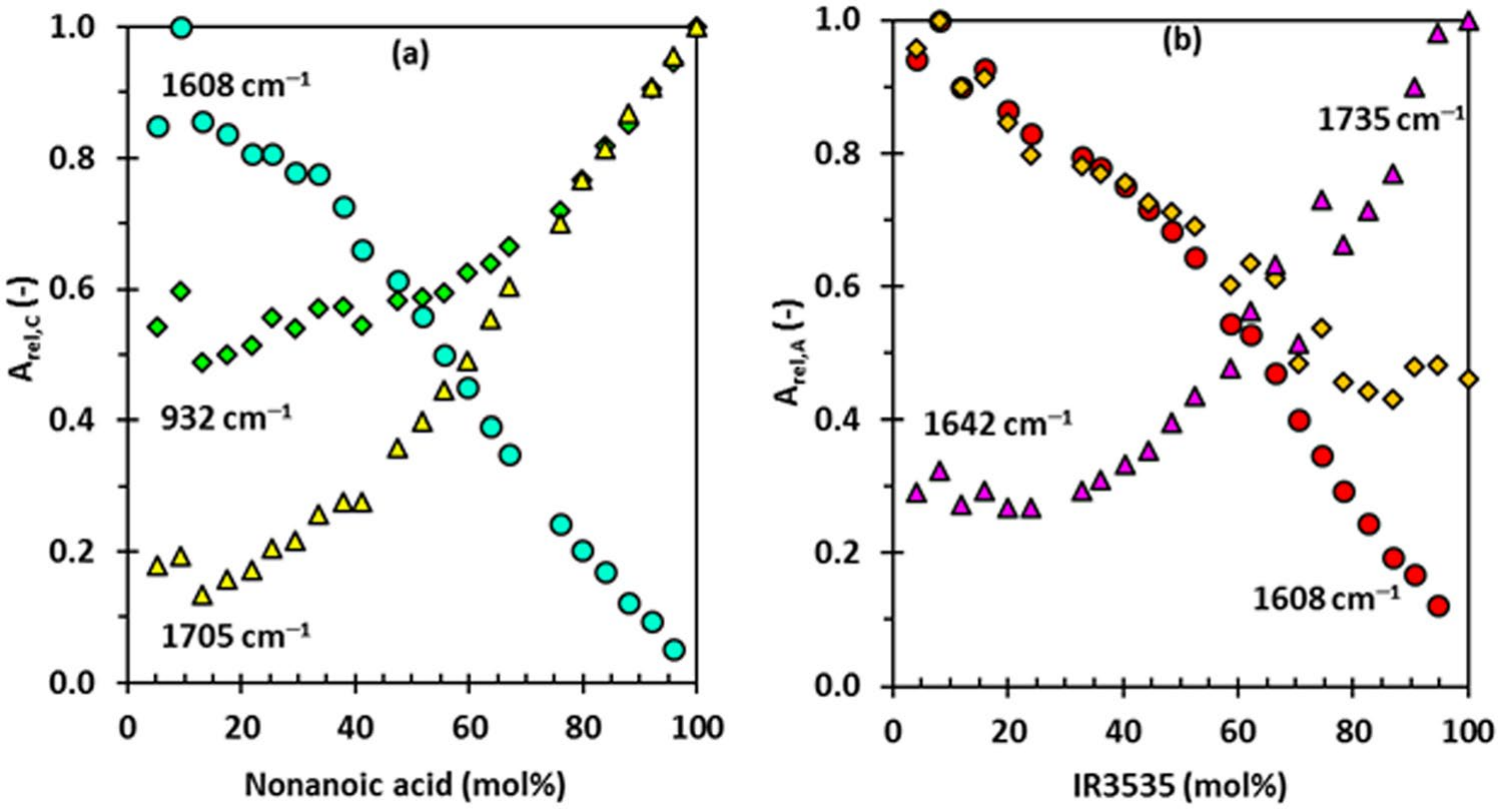
Effect of mixture composition on the relative carbonyl absorptions band intensities for (**a**) nonanoic acid, and (**b**) IR3535.

It is interesting to visualise the trends in the change in the intensity of absorption relative to the fraction of molecules present in the mixture (see Supplementary Information (SI. 4). Figure 7 shows plots for the trends in the relative absorbance for the carbonyl moieties in the present mixtures. Consider that the 932 cm^−1^ band is unique to the carboxylic acid dimers but that the 1705 cm^−1^ band is a composite representing all acid carbonyls, i.e. those present in monomers, dimers and linear and other cyclic oligomers.

Figure 7a shows that these bands initially exhibit an in-tandem, linear decrease in intensity as the acid content of the mixture is lowered. However, as the acid approaches the equimolar content, the decrease in relative dimer content levels off, while the band characteristic of all the species continues to decrease. On the other hand, the carbonyl peak characteristic for the hydrogen bonding interaction of the acid with the amide group continues to intensify as the acid content is depleted. Therefore, at a low acid concentration, the dimer acid and the acid-amide hydrogen-bonded forms are favoured above other forms, and in particular the monomers. This could explain the formation of the negative pseudo-azeotrope.

Figure 7b shows the corresponding trends for the carbonyl peaks associated with the IR3535 molecule. Here also, the 1608 cm^−1^ carbonyl peak, characteristic for the hydrogen bonding interaction of the carboxylate carbonyl with the amide group, continues to intensify as the IR3535 content decreases. This shows that it is a preferred intermolecular interaction. Such a band intensity trend is also shown by the ester carbonyl. Also, the peak position of the absorption band simultaneously shifts to higher wavenumbers. These observations probably indicate that this functional group experiences weaker intermolecular interactions in dilute solutions. The relative intensity of the amide band (1642 cm^−1^) also decreases as the IR3535 concentration decreases, but it also approaches a plateau value in the dilute region. Viewed together, these observations suggest that, in the dilute region, the aggregation of IR3535 molecules is reduced and that monomer forms are present. This provides a rationalisation for the higher vapour pressures that the mixtures exert near the positive pseudo-azeotrope composition.

## Molecular modelling

**Figure 8:**
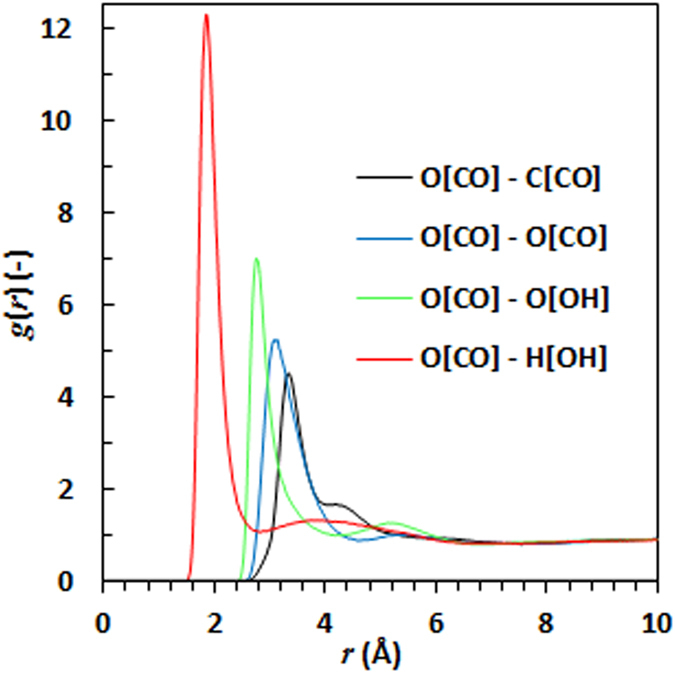
Partial radial distribution functions of the carbonyl oxygen atom in the nonanoic acid molecule.

Several investigators have studied the structure of carboxylic acids in the liquid state^16, 17^. Radial distribution functions of nonanoic acid, depicted in Fig. 8, provide quantitative information on the spatial intermolecular correlations present between the acid’s constituent atoms. The strong peak at 1.8 Å in the radial distribution function of the carbonyl oxygen pair in nonanoic acid is attributed to hydrogen bond formation^17^. The formation of strong hydrogen-bonded cyclic dimers is indicated by the expected orientation of the molecules in conjunction with the position of the other peaks^17^. Moreover, deconvolution of the radial distribution function involving hydroxyl hydrogen and hydroxyl oxygen, discloses three distinct peaks around 3.3 Å, 3.9 Å and 4.6 Å (see Supplementary Information (SI. 5). The first peak corresponds to the cyclic dimer, while the second and third peaks are attributed to the presence of higher-order aggregates in the liquid^18^.

The presence of two different polar functional groups in the chemical structure of IR3535, i.e. an amide and an ester, facilitates several complicated intramolecular and intermolecular interactions. There are four possible hydrogen bonds that can form in IR3535: (1) a carbonyl oxygen of the ester group and an aliphatic hydrogen; (2) a carbonyl oxygen of the amide group and an aliphatic hydrogen; (3) an sp^3^ oxygen of the ester group and an aliphatic hydrogen; and (4) a nitrogen and an aliphatic hydrogen. All these interactions can be either intra- or intermolecular. These multiple possibilities complicate the interpretation of the interactions in pure IR3535.

**Figure 9:**
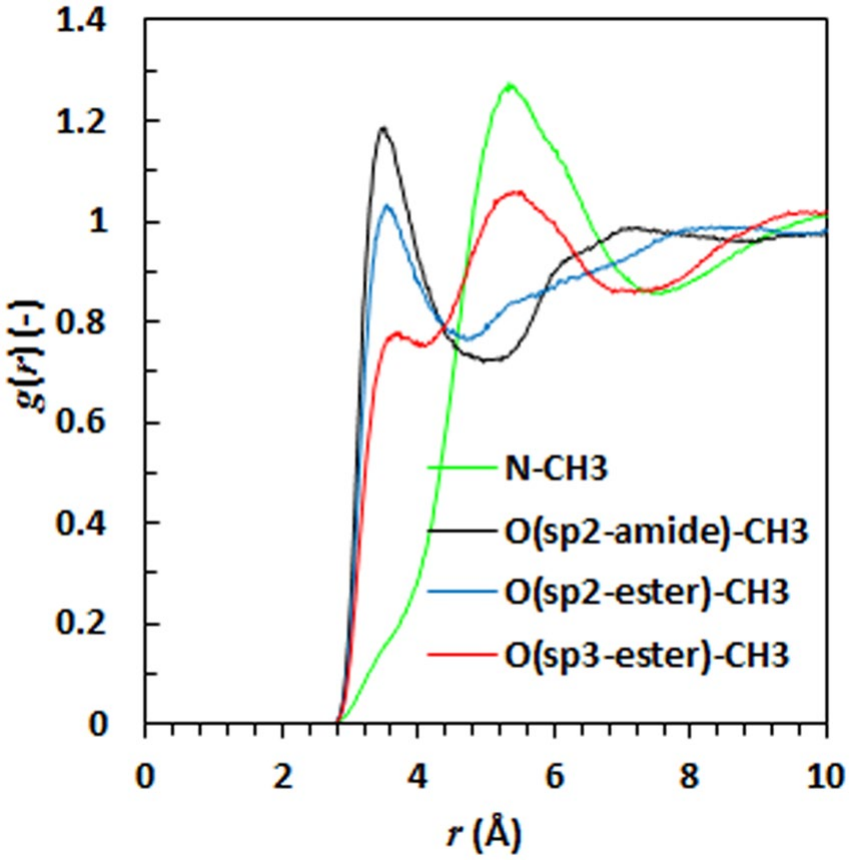
Partial radial distribution functions of either nitrogen or oxygen with methyl groups in IR3535.

The partial RDFs involving the methyl groups (and not other aliphatic carbons) and the hydrogen bond acceptors, either oxygens or nitrogen, in IR3535 are presented in Fig. 9. It indicates a weak correlation between all the acceptors and the methyl group hydrogens. This is attributed to “soft hydrogen bonding”, an interaction that occurs between strong acceptors and C–H^19^. Although the soft hydrogen bond is considered to be a very weak intermolecular interaction, it plays a role in the fields of supramolecular chemistry and macromolecules^19^. This soft hydrogen bonding, together with other intermolecular nonbonding interactions that result from electrostatic or dipole/dipole interactions, is probably responsible for the clustering of IR3535 molecules. Two examples of IR3535 molecule aggregations are presented in Supplementary Information (SI. 6). Typically, the IR3535 molecules aggregate as triplets.

Radial distribution correlation analysis of neat nonanoic acid and neat IR3535 suggests that both compounds are strongly structured liquids in their pure form. This finding may explain the remarkable difference in the boiling point of these compounds compared with structural analogues (see Supplementary Information (SI. 7). The implication is that intermolecular interactions and molecular aggregations must be responsible for the significantly lower vapour pressures and higher boiling points of nonanoic acid and IR3535 compared to structurally similar compounds.

**Figure 10:**
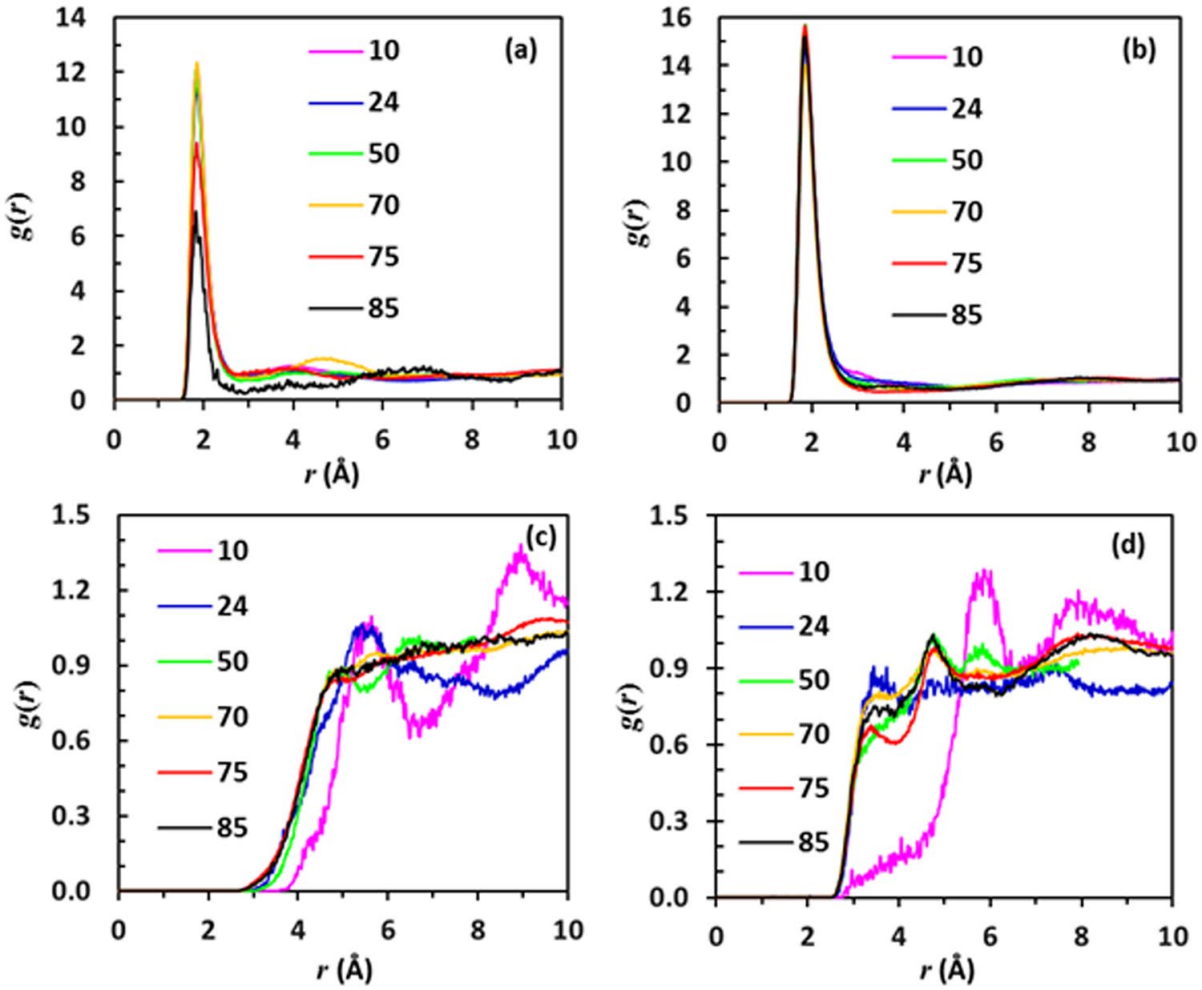
Partial radial distribution functions (**a**). H(OH) - O(CO) in the nonanoic acid molecule (**b**). H(OH) - O(sp^2^-amide) (**c**). O(sp^2^-ester) – N (**d**). O(sp^2^-amide) - O(sp^3^-ester) at various molar concentrations of IR3535. The indicated concentrations refer to the IR3535 content of the liquid phase.

Adding nonanoic acid to IR3535 brings about a rearrangement in the structuring of both compounds because of different intermolecular interactions. The modelling data indicate that the structuring of the liquid mixtures depends on the relative concentrations of the two components. The partial radial distribution functions involving the hydroxyl hydrogen and the carbonyl oxygen of nonanoic acid in a series of mixtures, presented in Fig. 10a, indicate that hydrogen-bonded dimer rings are still present even at 85 mol% IR3535.

Figure 10b shows the partial radial distribution functions for the hydroxyl hydrogen in nonanoic acid and the carbonyl oxygen of the amide group in IR3535. The considerable intensity and invariance of the peak indicate a very high propensity for hydrogen bond formation between the amide group and the hydroxyl hydrogen. This peak is much more pronounced than the peaks for any other group. The peak’s maximum is reached at ca. 77 mol% IR3535, i.e. near the negative pseudo-azeotrope composition.

The snapshots generated by MedeA^®^-GIBBS Version 2.18 software indicate that at a high concentration of IR3535, most of the nonanoic acid molecules interact with the IR3535 present. A snapshot obtained at the molar ratio of 3:1, reveals the presence of large clusters of IR3535-nonanoic acid (see Supplementary Information (SI. 8). This extensive molecular aggregation provides a likely explanation for the significantly reduced volatility of the binary mixture at this concentration.

Figure 10c shows that the distance between the ester carbonyl oxygen and nitrogen increases with increasing acid content. This suggests that the IR3535 molecules tend to become separated from each other. This is most clearly shown in Fig. 10d where an increase in the separation between the ester and the amide functional group is evident. The loss of aggregation between the IR3535 molecules in the presence of larger amounts of nonanoic acid is the probable cause of the increased volatility at the positive pseudo-azeotrope composition of ca. 10 mol% IR3535 in the binary mixture.

## Conclusion

The evaporation behaviour of mixtures of the known mosquito repellents IR3535 and nonanoic acid was studied. Mass loss measurements combined with spectroscopic tracking of changes in composition revealed the presence of two pseudo-azeotrope compositions. A positive and a negative azeotrope are located at ca. 10 and 77 mol% IR3535 respectively. The latter mixture features the lowest vapour pressure, lower than that of the two neat repellents. This combination provided excellent mosquito repellence when tested as a topical application. It was significantly more effective, with repellence persisting for up to four hours. It also exhibited a strong knock-down effect that even caused partial mosquito mortality.

The presence of two pseudo-azeotrope points at different compositions in a binary system is a rare occurrence. Molecular simulation techniques were therefore used to explore the nature of the system and the interactions responsible for this unique behaviour. Gibbs-Monte Carlo simulation results suggest that variations in the sizes of the molecular clusters present in the liquid at various compositions might be responsible. They revealed that IR3535 and nonanoic acid in neat form are both highly structured liquids. The breakdown in the structure of IR3535 at high concentrations of the acid may be the origin of the increased evaporation rate and the formation of the positive pseudo-azeotrope. On the other hand, a negative pseudo-azeotrope may result from the absence of acid monomers and the formation of larger molecular clusters at the ratio of IR3535 to nonanoic acid of 3:1. This approach will open the way for the development of better mosquito repellent formulations based on azeotropic blends, which are sorely needed to fight mosquito-borne diseases.

## Methods

### Material selection

Ethyl butylacetylaminopropionate (IR3535) [CAS Number: 52304-36-6] was identified as a safe and effective mosquito repellent^20^. It features an ester and a tertiary amide functional group, both of which can act as hydrogen bond acceptors. Carboxylic acids are capable of forming strong interactions with such groups. A range of carboxylic acids, which are molecules capable of forming strong interactions with IR3535, was reviewed. Nonanoic acid [CAS Number: 112-05-0] was selected on the basis of safety^21^, its intrinsic pH as a proxy for skin irritation potential and its inherent repellence effect^22^. IR3535 (>98%) was supplied by Merck Chemicals and nonanoic acid (>96%) was obtained from SigmaAldrich. The reagents were used as received without further purification.

### Oven test

The typical sign of azeotropic mixtures is the constant composition during equilibrium distillation. However, the process involved in the application of repellents is open evaporation and not distillation. A constant composition would substantiate the presence of a pseudo-azeotrope composition during evaporation. Although evolved gas analysis such as TG-FTIR can be used to investigate the evaporation mechanism of liquids, the volatility of the present mixtures, was too low to allow sensing the vapour by the coupled identification techniques. We, therefore, developed an oven test to assess the constant releasing behaviour, i.e. to confirm the presence of pseudo-azeotropic mixtures and establish their compositions.

Fourteen different binary mixtures of IR3535 and nonanoic acid were prepared at various concentrations. The samples were transferred to evaporation dishes. The volume of the mixture in each evaporation dish was 13.5 ± 0. 1 mL. All the samples were placed on a rotating table inside a temperature-controlled convection oven. Also, a wire mesh was installed in front of the convector to help homogenise the air flow to different areas of the oven. This setup evened out the effects of variations in the resident air flow patterns, i.e. all the dishes experienced identical environment conditions. The evaporation dishes were balanced on the rotating table after each measurement. The test was conducted at 50 ± 1 °C.

At certain time intervals, the mass loss of the mixture was recorded using an electronic balance (XPS360 model with 0.001 g accuracy). At these times, 10 µL samples of the mixtures were taken and used to record FTIR spectra. The sample mass was recorded again after each FTIR measurement to correct for the material loss caused by the sampling. The liquid-phase FTIR spectra were recorded at room temperature in the wavenumber range 4000 to 600 cm^−1^ at a resolution of 2 cm^−1^. The liquid in the dishes was allowed to evaporate continuously, and samples for FTIR were removed periodically for a total of approximately one month. The composition of the liquid samples was determined from the recorded FTIR spectra using an inverse technique.

### The inverse composition identification method

The liquid compositions were determined by an inverse mapping approach. A surrogate model of the inverse system was constructed to map the measured system response to the system parameters directly^23^. Partial least squares regression (PLSR) was used to construct the inverse map^15, 24^. A set of 24 different mixtures of IR3535 with nonanoic acid of known composition was prepared. These mixtures provided the training set for the known test variable (composition) and response matrices (FTIR spectra).

Cross-validation was used to determine the optimum number of PLSR directions. This was done by sequentially removing one of the 24 training points from the training set and using it as a validation point. The PLSR was then constructed using the remaining 23 training points to estimate the composition of the validation point from its spectrum. This process was repeated, in turn, for all 24 training points. The mean errors for the PLSRs constructed with from 1 to 15 PLSR directions were computed. The optimum number of PLSR directions was determined to be three. For this choice the maximum- and the mean prediction errors for composition were 2.1 mol% and 0.5 mol% respectively.

### Thermogravimetric analysis (TGA)

TGA was used as the standard technique to measure the vapour release rate of the liquid mixtures. The measurements were performed on a Perkin-Elmer TGA 4000 thermogravimetric analyser. A 180 μL open alumina pan was partially filled with 85 ± 5 mg of sample. The sample was kept isothermal at 50 °C for the duration of the test period. The highest temperature at which repellents are expected to function during actual commercial use, i.e. 50 °C was selected to maximise volatility. The data were collected under nitrogen (N_2_) at a flow rate of 50 mL min^−1^ to prevent oxidation.

### Molecular modelling

Monte Carlo simulations were performed using the MedeA^®^-GIBBS Version 2.18 software suite in the isothermal-isobaric ensemble (NPT). The number of molecules, the temperature and the pressure were kept constant at respectively 250, 323 K and 1 bar throughout the simulations, while the density of the systems was allowed to fluctuate.

Both the nonanoic acid and the IR3535 molecules were treated as semi-flexible molecules. Different types of moves with appropriate probabilities were implemented taking into account the type of ensemble, i.e. NPT, and the choice of semi-flexible molecules.

The anisotropic united-atom (AUA) force field was used to describe the intermolecular forces and intermolecular potential energy. It was necessary to add parameters in order to describe the potentials in AUA for tertiary amides. The modified AUA force fields were validated using a set of tertiary amides which included IR3535. The additional parameters are presented in the Supplementary Information (SI. 9).

### Repellence

#### Ethical approval

Ethical approval for this research was granted by *The Faculty of Health Sciences Research Ethics Committe*e of the University of Pretoria via Record number 26/2016. The mosquito repellence test protocols employed were authorised by the Health Ethics Committee of the South African Medical Research Council (MRC). All the experiments were performed in accordance with relevant guidelines and regulations enforced by the MRC. Four persons participated in the MRC tests conducted at the Medical Research Council facilities in Durban, South Africa (MRC). Informed consent was obtained from all four human subjects.

#### Animal preparation

The standard WHO guidelines were adopted for use in this trial^11, 25^. Adult female *Anopheles arabiensis* mosquitoes were collected from a stock population cage in which both sexes were kept. The mosquitoes were maintained at a temperature of 27 °C and a relative humidity of 70% under 12/12-hour light/dark photo periods. Adults were provided with 10% sucrose solution and were periodically blood-fed on restrained guinea pigs.

Repellence assays were performed with 3- to 5-day-old *An*. *arabiensis* females that had been starved for six hours, but which had previously had access to a 10% sucrose solution. Repellent activity was assessed by topical application of the test substance to the skin on human arms. Subsequently, the treated area was exposed to the unfed female mosquitoes. According to the WHO protection guide, (*P*) is stated in terms of the number of mosquitoes probing on the treated arm (*T*) relative to the number probing on the control arm (*C*), according to the formula:

$$P=1-T/C$$

#### Test procedure

Paper cups (500 mL) were modified by replacing the base of the cup with mosquito netting that was held in place with a rubber band. The mouth of the cup was covered with transparent plastic film. Twenty unfed 3- to 5-day-old, active host-seeking *An*. *Arabiensis* females, selected from the test mosquitoes via an aspirator, were introduced to the cup. All the repellents were tested on two male and two female volunteers. The test area of the volunteers’ skin was first washed with unscented soap and rinsed with water. The repellent formulation (0.2 mL) was applied evenly to 5 cm^2^ on the test forearm, and 0.2 mL of acetone was applied to the control arm and left to dry. Initially, the readiness of the mosquitoes to probe was assessed by placing an untreated arm in contact with the cup for 30 seconds. Mosquito activity was observed through the transparent plastic film. In the next step, the cup was kept in contact with the control and treated forearms of the volunteers. During the 3 min exposure period, the number of mosquitoes probing (attempting to feed on the volunteers through the netting) was recorded. The repellence effect was determined hourly for up to six hours. For each measurement, a new batch of mosquitoes was introduced to the same cup.

### Statement on the use of human participants

Ethical approval for this research was granted by *The Faculty of Health Sciences Research Ethics Committe*e of the University of Pretoria. Four persons participated in the MRC mosquito repellence tests conducted at the Medical Research Council in Durban, South Africa (MRC). The protocols implemented have full MRC approval and and these tests are conducted on a routine basis for external customers and researchers. We hereby confirm that all the experiments were (a) performed in accordance with relevant guidelines and regulations enforced by the MRC, and (b) that informed consent was obtained from all subjects.

## Electronic supplementary material

Supplementary Information
