# Supplementary material for: A promising azeotrope-like mosquito repellent blend
Source: Sci Rep. 2017 Aug 31;7:10273. doi: 10.1038/s41598-017-10548-y (PMC5579307; doi:10.1038/s41598-017-10548-y)
Supplement: Supplementary file 1 — Supplementary Information [file 41598_2017_10548_MOESM1_ESM.pdf]

A promising azeotrope-like mosquito repellent blend

Homa Izadi[1,2], Walter W Focke[1,2,*], Erfan Asaadi[1], Rajendra Maharaj[2,3], Jannie Pretorius[4] and Mattheüs Theodor Loots[5]

Supplementary Information

SI. 1. **The physical concept behind formation of pseudo-azeotropes**

Raoult's law states that the equilibrium vapour pressure of an ideal liquid vaporising as an ideal gas is simply the mole fraction weighted mean of the vapour pressures of the neat components. i.e.

$$P^{vap} = x_A P_A^{sat} + x_B P_B^{sat} \qquad \textbf{(SI. 1. 1)}$$

where P^{vap} is the vapour pressure exerted by the mixture while x_i and P_i^{sat} are the liquid composition and vapour pressure of component *i* respectively. For real liquids, the activity coefficients γ_i must be considered and the revised equation is:

$$P^{vap} = x_A \gamma_A P_A^{sat} + x_B \gamma_B P_B^{sat} \qquad \textbf{(SI. 1. 2)}$$

When the activity coefficients assume values below unity, this equation predicts that the vapour pressure of the mixture will be less than the value predicted by Raoult's law. That is generally the case when the strength of the attractive interactions between the unlike molecules exceed those between the like molecules in the liquid mixture. When these attractive interactions between the unlike molecules are sufficiently strong, the predicted liquid vapour pressure curve may even dip below the vapour pressures of the two parent compounds. The location of the resulting minimum in the vapour pressure curve represents a negative pseudo-azeotrope composition since the observed vapour pressure will be below the value predicted by Raoult's law.

SI. 2. **Description of the spectral features of nonanoic acid and IR3535 FTIR spectra**

The spectrum for the nonanoic acid has, among others, the following distinguishing features. The strongest peak, located at 1706 cm^{-1}, arises from the carbonyl stretch vibration. This peak is actually a composite of several overlapping absorptions contributed by the carboxylic acid moiety. In order from high to lower wavenumbers, it includes contributions from the terminal groups of linear dimers and polymers, monomers, the cyclic dimer and the inner groups of linear dimers/oligomers[1]. A very broad peak for the nonanoic acid is centred approximately at 3000 cm^{-1}. It is superimposed on the C–H stretch bands, but it is much broader, having a half-intensity band width of 700 cm^{-1} [2]. The unusual width and shape of the O–H stretching band of carboxylic acids arises, in part, from the presence of an equilibrium mixture of open chain and cyclic carboxylic acid forms[3]. The band at ca. 932 cm^{-1}, is known as the "dimer band"[4] as it occurs only for dimer carboxylic acid structures. It arises from concerted out-of-plane motions of the two hydrogens in the dimer ring[5]. The intermolecular interaction in the mixture of IR3535 and nonanoic acid results in the development of a new peak in the FTIR spectra located at 1608 cm^{-1}. The position of this peak appears to be almost independent of mixture composition.

SI. 3. **The spectral residuals for IR3535-nonanoic acid binary mixtures**

Spectral residual amplifies differences arising from intermolecular interactions. ΔA_{mix} is defined by: $\Delta A_{mix} = A_{mix} - x_A A_A - x_C A_C$ where A_{mix} is the absorbance measured for the actual mixture with compositions corresponding to mole fractions x_A and x_C; the subscripts A and C denoting IR3535 and nonanoic acid respectively. If the interactions the molecules experience in the mixture were identically the same as in the neat liquids, then ΔA_{mix} would be exactly zero. However, this is not the case here. Some bands are characteristic of functional groups present on only one of the constituents. Others arise from the interactions between the unlike molecules. In the former case, negative values for ΔA_{mix} associated with a given band indicates that, compared to the situation in the neat liquid, the effect of the molecular vibration is less than expected.

SI. 4. **The relative absorptions band intensities for nonanoic acid-IR3535 binary mixtures**

To calculate the intensity of absorption relative to the fraction of molecules present in the mixture, it is more helpful to consider the relative changes in the peak intensities in the following way:

$$A_{rel,A} = \left(A_{mix} - x_C A_C\right)/\left(x_A A_{\max,A}\right) \quad \text{and} \quad A_{rel,C} = \left(A_{mix} - x_A A_A\right)/\left(x_C A_{\max,C}\right)$$

These variables should assume values of unity if the absorption band characteristic for the given compound under investigation is not affected by the presence of the other compound.

SI. 5. **Deconvolution and curve- fitting of the peak related to H(OH)-O(OH) in partial radial distribution function of nonanoic acid**

The peak is deconvoluted to Curve 1 (3.3 Å), Curve 2 (3.9 Å), and Curve 3 (4.6 Å) and curve-fitted on Fit Sum. The first peak corresponds to the cyclic dimer while the second peak is attributed to the presence of higher order aggregates in the liquid[6]. Partial radial distribution of H(OH)-O(OH) reveals an interesting aspect of the hydrogen bonding in nonanoic acid. Although there are two oxygen atoms capable of forming hydrogen bonds in the nonanoic acid molecule[6], only the carbonyl oxygen is involved. This is reflected in absence of any major peak at short distances, around 1.8 Å, i.e. where hydrogen bonds usually peak.

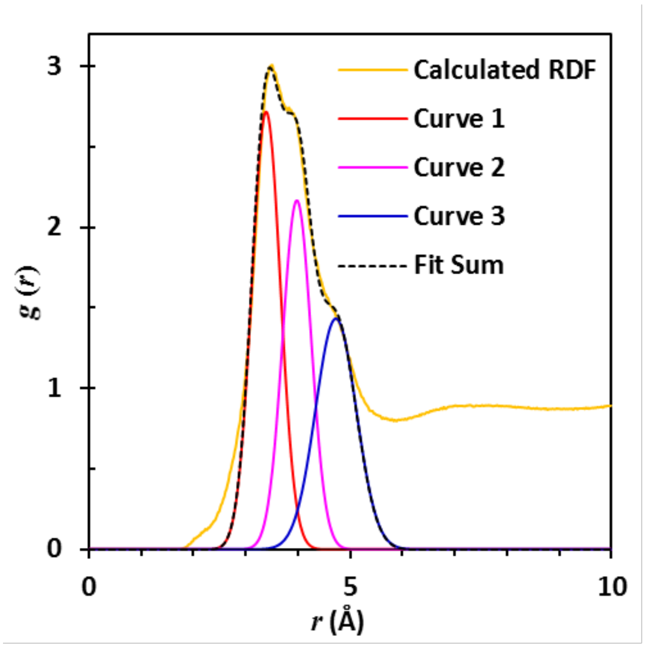

Deconvolution and curve- fitting of the peak related to H(OH)-O(OH) in partial radial distribution function of nonanoic acid as calculated presently.

SI. 6. **Typical aggregations of IR3535 molecules**

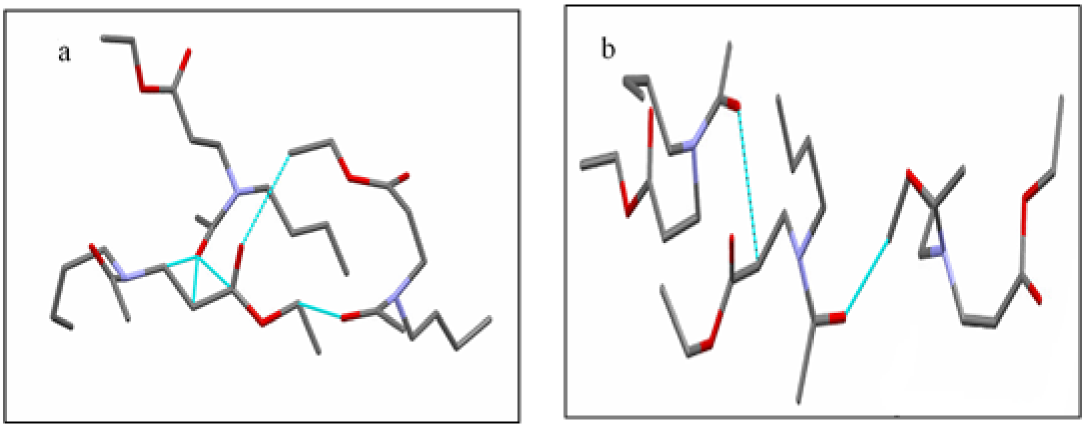

Two typical aggregations of IR3535 molecules. Red, blue, and grey lines represent oxygen, nitrogen and carbon atoms, respectively. The short contacts are shown with dash blue lines.

SI. 7. **Thermal properties of IR3535, nonanoic acid and their structural analogues**

Table SI.7.1. Chemical structures and boiling points of nonanoic acid and 2-Decanone

**Compound**	**Chemical structure**	**Boiling point (°C)** **(Condition: 760 Torr)**
Nonanoic acid **($C_9H_{18}O_2$)**	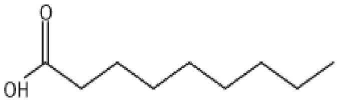	**254**[7]
2- Decanone **($C_{10}H_{20}O$)**	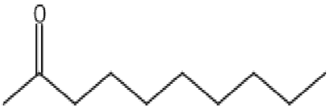	**210**[8]

Table SI. 7.2. Chemical structures and boiling points of IR3535 acid and Octanoic acid 4-acetyl- ethyl ester

**Compound**	**Chemical structure**	**Boiling point (°C)** **(Condition: 760 Torr)**	**Comments**
Ethyl butylacetylaminopropionate (IR3535) **($C_{11}H_{21}NO_3$)**	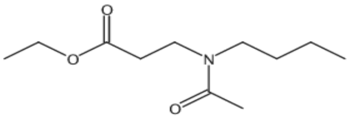	**314.8±25.0**	Calculated using Advanced Chemistry Development (ACD/Labs) Software V11.02 (© 1994-2016 ACD/Labs)
Octanoic acid, 4-acetyl-, ethyl ester **($C_{12}H_{22}O_3$)**	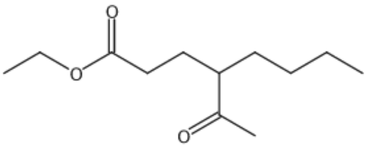	**290.0±13.0**	Calculated using Advanced Chemistry Development (ACD/Labs) Software V11.02 (© 1994-2016 ACD/Labs)

SI. 8. **IR3535-nonanoic acid (3:1) aggregation**

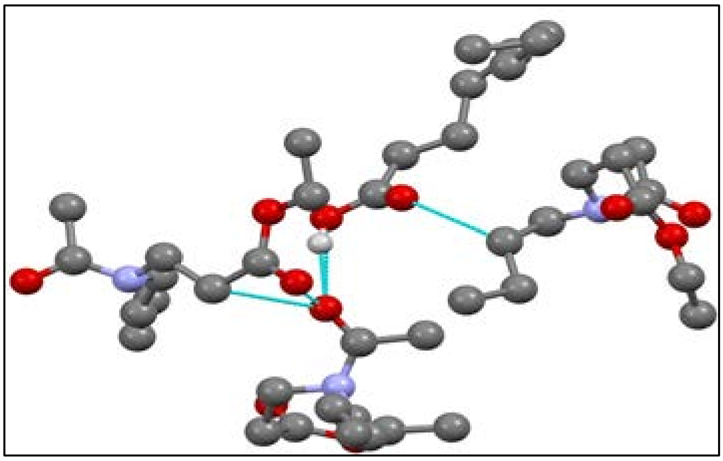

Snapshot of IR3535-nonanoic acid (3:1) aggregation at P = 1 atm, T = 323 K. Red, blue, and grey spheres represent oxygen, nitrogen and carbon atoms, respectively. The short contacts are shown with dash blue lines.

SI. 9. **Added parameters to AUA forcefield**

Table SI.9.1. Added stretching parameters of the anisotropic united atoms intermolecular potential for tertiary amides

	L_0	Ref.
$N_{amide} - C_{sp2,\ amide}$	1.3729 Å	[9]

Table SI.9.2. Added bending parameters of the anisotropic united atoms intermolecular potential for tertiary amides

	K	θ	Ref.
$C_{sp2,\ amide} - N_{amide} - CH3$	57128.00	111.0	[10,11]
$C_{sp2,\ amide} - N_{amide} - CH2$	57128.00	111.0	[10,11]
$N_{amide} - C_{sp2,\ amide} - O_{sp2,\ amide}$	114288.00	122.9	[12,13]
$N_{amide} - C_{sp2,\ amide} - CH2$	80438.00	119.2	[12,13]
$N_{amide} - C_{sp2,\ amide} - CH3$	80438.00	119.2	[12,13]
$O_{sp2,\ amide} - C_{sp2,\ amide} - CH2$	80438.00	120.4	[12,14]
$O_{sp2,\ amide} - C_{sp2,\ amide} - CH3$	80438.00	120.4	[12,14]
$CH3 - C_{sp2,\ amide} - CH3$	105822.00	119.2	[15,16]
$CH3 - C_{sp2,\ amide} - CH2$	105822.00	119.2	[15,16]
$CH2 - C_{sp2,\ amide} - CH2$	105822.00	119.2	[15,16]
$CH3 - CH2 - C_{sp2,\ amide}$	74900.00	114.0	[15,17]
$CH2 - CH2 - C_{sp2,\ amide}$	74900.00	114.0	[15,17]

Table SI.9.3. Added torsion parameters of the anisotropic united atoms intermolecular potential for tertiary amides (In units of K)

**A0**	**A1**	**A2**	**A3**	**A4**	**A5**	**A6**	**A7**	**A8**
CH3 – N_{amide} – $C_{sp2, amide}$ – CH3[10]								
189.01	979.05	482.82	-1436.8	-1465.1	-1219.3	913.04	541.13	16.99
CH3 – N_{amide} – $C_{sp2, amide}$ – CH2[10]								
189.01	979.05	1482.82	-1436.8	-1465.1	-1219.3	913.04	541.13	16.99
CH2 – N_{amide} – $C_{sp2, amide}$ – CH3[10]								
189.01	979.05	1482.82	-1436.8	-1465.1	-1219.3	913.04	541.13	16.99
CH2 – N_{amide} – $C_{sp2, amide}$ – CH2[10]								
189.01	979.05	1482.82	-1436.8	-1465.1	-1219.3	913.04	541.13	16.99
$O_{sp2, amide}$ – $C_{sp2, amide}$ – N_{amide} – CH3[12]								
468.4	-583.65	-210	842	0	0	0	0	0
$O_{sp2, amide}$ – $C_{sp2, amide}$ – N_{amide} – CH2[12]								
468.4	-583.65	-210	842	0	0	0	0	0
$C_{sp2, amide}$ – CH2 – CH2 – N_{amide}[10]								
816.65	2509.94	9.01	-3609	-54.51	286.01	-104.22	-133.18	279.1
CH2 – CH2 – N_{amide} – $C_{sp2, amide}$[10]								
189.01	979.05	1482.82	-1436.8	-1465.1	-1219.3	913.04	541.13	16.99
**A0**	**A1**	**A2**	**A3**	**A4**	**A5**	**A6**	**A7**	**A8**

Table SI.9.4. Applied charges for tertiary amides

	N_{amide}	CH2(-N)	CH3(-N)	$O_{sp2, amide}$	$C_{sp2, amide}$
**Charge**	-0.46	0.275	0.275	-0.45	0.45
**Ref.**	[18]	[18]	[18]	[15]	[15]

SI. 10. Regression of the oven evaporation test and repellence data

Non-linear regression, using the "nls" function in R[19] was used for fitting the models in this section. For the oven evaporation test (Table SI.10.1), the Avrami model was fitted to the data obtained for starting IR3535 concentrations of 40 mol% and higher:

$$\frac{x(\ell)-x_{azeo}}{x_o-x_{azeo}}=exp\left[-\left(\frac{\ell}{\lambda}\right)^n\right] \qquad \textbf{(SI.10.1)}$$

where $x(\ell)$ is the concentration (mol%) of IR3535 in the remaining liquid at the point where a portion ℓ of it has evaporated; x_{azeo} is the pseudo azeotrope composition; x_o is the initial concentration IR3535 in the liquid; λ and n are model parameters. This model was explicitly coded with initial values corresponding to manual guesses. The logistic function was fitted to the repellence data (Table SI.10.3) using the self-starting "SSlogis" function:

$$Proportion=\frac{S}{1+exp\left[\left(c-t\right)/\tau\right]} \qquad \textbf{(SI.10.2)}$$

where the proportion refers to the protection rendered by the repellent in the test at time t. The parameter S can be associated with an estimate for the highest protection possible c is a shift parameter and τ provides a rough measure of the maximum time duration of protection.

In both cases, the "nlsBoot" function from the "nlstools" package[20] was used in order to obtain non-parametric bootstrap confidence intervals for all model parameters[21]. The bootstrap sample was set to 5,000 in all cases, but may effectively be less because of incomputable sample combinations.

Table SI.10.1 Oven test data. Liquid composition ($x(\ell)$) expressed as mol% IR3535 *vs*. the mass% liquid that has evaporated (ℓ)

ℓ	0.00	15.28	30.50	45.46	59.95	74.51	88.87		
$x(\ell)$	10.01	10.08	10.00	10.51	10.41	10.18	10.15		
ℓ	0.00	13.25	24.40	36.37	48.06	58.54	69.46	80.90	92.51
$x(\ell)$	15.09	16.58	18.04	19.55	21.74	23.19	25.27	28.19	35.42
ℓ	0.00	12.69	23.32	33.47	43.79	53.68	63.28	74.52	85.38
$x(\ell)$	20.14	22.29	24.85	26.55	28.92	32.06	36.01	44.86	47.15
ℓ	0.00	10.18	19.93	29.14	38.91	48.36	57.92	67.26	76.51
$x(\ell)$	25.71	28.04	30.60	32.92	35.17	37.55	41.55	47.25	52.11
ℓ	0.00	10.47	20.41	29.56	38.79	48.21	57.82	67.05	76.46
$x(\ell)$	30.02	33.02	36.28	39.55	42.24	45.51	49.05	54.01	57.86
ℓ	0.00	10.74	20.17	29.85	39.15	48.59	58.16	67.22	76.89
$x(\ell)$	40.20	44.83	48.18	51.67	55.95	61.05	66.27	70.05	72.08
ℓ	0.00	10.71	20.65	30.40	39.94	49.72	56.72	65.44	71.34
$x(\ell)$	50.80	54.94	58.47	62.87	66.55	69.51	70.55	71.25	72.15
ℓ	0.00	9.48	18.99	28.39	36.54	44.45	52.51	59.56	66.69
$x(\ell)$	60.06	62.19	64.56	66.60	68.51	70.55	71.51	72.92	74.55
ℓ	0.00	9.26	18.36	25.61	31.70	37.31	42.56	48.44	53.80
$x(\ell)$	70.64	71.82	73.08	73.72	74.15	74.60	74.99	74.80	75.21
ℓ	0.00	5.30	10.67	16.05	21.36	26.67	32.02	37.29	42.56
$x(\ell)$	75.25	75.21	75.25	75.37	75.51	75.77	75.95	75.99	76.14
ℓ	0.00	7.33	13.84	20.04	25.23	30.58	35.85	41.27	46.58
$x(\ell)$	80.99	79.59	78.30	77.05	77.63	77.20	77.08	76.90	76.80
ℓ	0.00	8.83	17.63	26.31	35.23	44.19	54.59	62.91	70.51
$x(\ell)$	90.14	89.30	88.44	87.31	85.56	83.55	80.91	79.03	77.51

Table SI.10.2 Bootstrap statistics for Oven Test Data

Parameter	Estimate	Std. error	Median	2.5%	97.5%
n	1.4868	0.074743	1.4842	1.348	1.6397
λ	48.2749	1.190904	48.2699	46.044	50.5950
x_{azeo}	76.7112	0.402963	76.7128	75.879	77.4594

Table SI.10.3 Oven test data. Liquid composition ($x(\ell)$) expressed as mol% IR3535 *vs*. the mass liquid that has evaporated (ℓ)

		Time (min)						
**Repellent**	**#**	**3**	**30**	**60**	**120**	**180**	**240**	**360**
DEET	1	0.92	0.67	1.00	1.00	0.25	0.00	0.00
DEET	2	0.79	0.64	0.93	0.86	0.29	0.00	0.00
DEET	3	0.86	0.64	0.93	0.93	0.14	0.00	0.00
DEET	4	0.76	0.65	0.71	0.76	0.29	0.00	0.00
IR3535	1	0.67	0.67	0.42	0.42	0.33	0.58	0.33
IR3535	2	0.71	0.86	0.79	0.64	0.50	0.50	0.14
IR3535	3	0.64	0.71	1.00	0.79	0.79	0.79	0.79
IR3535	4	0.65	0.71	0.65	0.65	0.59	0.65	0.47
Blend	1	1.00	1.00	1.00	1.00	1.00	1.00	0.67
Blend	2	1.00	1.00	1.00	1.00	1.00	1.00	0.64
Blend	3	1.00	1.00	1.00	1.00	1.00	1.00	0.64
Blend	4	1.00	1.00	1.00	1.00	1.00	1.00	1.00

Table SI.10.4 Bootstrap statistics for repellence data

Repellent	Parameter	Estimate	Std. error	Median	2.5%	97.5%
**DEET**	S	0.81738	0.024237	0.8173	0.76921	0.86598
	c	177.15073	4.781167	179.3997	162.95279	180.09179
	τ	-3.77620	5.331984	-1.1243	-18.64460	-0.39182
**IR3535**	S	0.92289	1.0286	0.73799	0.6239	2.3691
	c	323.52325	252.9073	369.84577	-398.0891	563.1272
	τ	168.50401	150.1500	-124.36639	-575.4869	-3.0677
**Blend**	S	0.9998	0.0109	0.99919	0.97905	1.0246
	c	365.3741	8.8343	363.42512	361.4260	391.5035
	τ	-5.5243	7.8248	-3.70079	-29.32209	-1.5934

References

1 Ng, J. B. & Shurvell, H. F. Application of factor analysis and band contour resolution techniques to the Raman spectra of acetic acid in aqueous solution. *The Journal of Physical Chemistry* **91**, 496-500, doi:10.1021/j100286a046 (1987).

2 Bratož, S., Hadži, D. & Sheppard, N. The infra-red absorption bands associated with the COOH and COOD groups in dimeric carboxylic acid—II: The region from 3700 to 1500 cm−1. *Spectrochimica Acta* **8**, 249-261, doi:http://dx.doi.org/10.1016/0371-1951(56)80031-3 (1956).

3 Bellamy, L. J., Lake, R. F. & Pace, R. J. Hydrogen bonding in carboxylic acids—II. Monocarboxylic acids. *Spectrochimica Acta* **19**, 443-449, doi:http://dx.doi.org/10.1016/0371-1951(63)80056-9 (1963).

4 Mayo, D. W., Miller, F. A. & Hannah, R. W. *Course notes on the interpretation of infrared and Raman spectra*. (Wiley Online Library, 2004).

5 Hadzi, D. & Sheppard, N. The Infra-Red Absorption Bands Associated with the COOH and COOD Groups in Dimeric Carboxylic Acids. I. The Region from 1500 to 500 cm^{-1}. *Proceedings of the Royal Society of London. Series A. Mathematical and Physical Sciences* **216**, 247-266, doi:10.1098/rspa.1953.0020 (1953).

6 Xu, W. & Yang, J. Computer Simulations on Aggregation of Acetic Acid in the Gas Phase, Liquid Phase, and Supercritical Carbon Dioxide. *The Journal of Physical Chemistry A* **114**, 5377-5388, doi:10.1021/jp100040j (2010).

7 Golovanov, I. & Zhenodarova, S. Quantitative structure-property relationship: XXVI. Toxicity of aliphatic carboxylic acids. *Russian journal of general chemistry* **76**, 40-44 (2006).

8 Degani, I., Fochi, R. & Regondi, V. Mercury (II) oxide/35% aqueous tetrafluoroboric acid/tetrahydrofuran: an improved reagent for the hydrolysis of cyclic hemithioacetals, dithioacetals, and ortho esters. *Synthesis* **1981**, 51-53 (1981).

9 Aparicio-Martínez, S. & Balbuena, P. B. On the properties of aqueous amide solutions through classical molecular dynamics simulations. *Molecular Simulation* **33**, 925-938, doi:10.1080/08927020701474422 (2007).

10 Orozco, G. A., Nieto-Draghi, C., Mackie, A. D. & Lachet, V. Transferable force field for equilibrium and transport properties in linear and branched monofunctional and multifunctional amines. II. Secondary and tertiary amines. *The journal of physical chemistry. B* **116**, 6193-6202, doi:10.1021/jp302972p (2012).

11 Chen, K. H., Lii, J. H., Fan, Y. & Allinger, N. L. Molecular mechanics (MM4) study of amines. *Journal of computational chemistry* **28**, 2391-2412, doi:10.1002/jcc.20737 (2007).

12 Boutard, Y. *et al.* Extension of the anisotropic united atoms intermolecular potential to amines, amides and alkanols: Application to the problems of the 2004 Fluid Simulation Challenge. *Fluid Phase Equilibria* **236**, 25-41, doi:http://dx.doi.org/10.1016/j.fluid.2005.06.009 (2005).

13 Jorgensen, W. L., Maxwell, D. S. & Tirado-Rives, J. Development and Testing of the OPLS All-Atom Force Field on Conformational Energetics and Properties of Organic Liquids. *Journal of the American Chemical Society* **118**, 11225-11236, doi:10.1021/ja9621760 (1996).

14 Kranias, S., Pattou, D., Levy, B. & Boutin, A. An optimized potential for phase equilibria calculation for ketone and aldehyde molecular fluids. *Physical Chemistry Chemical Physics* **5**, 4175-4179, doi:10.1039/B304531E (2003).

15 Ferrando, N., Lachet, V. & Boutin, A. Monte Carlo Simulations of Mixtures Involving Ketones and Aldehydes by a Direct Bubble Pressure Calculation. *The Journal of Physical Chemistry B* **114**, 8680-8688, doi:10.1021/jp1031724 (2010).

16 Cornell, W. D. *et al.* A Second Generation Force Field for the Simulation of Proteins, Nucleic Acids, and Organic Molecules. *Journal of the American Chemical Society* **117**, 5179-5197, doi:10.1021/ja00124a002 (1995).

17 Ungerer, P. *et al.* Optimization of the anisotropic united atoms intermolecular potential for n-alkanes. *The Journal of Chemical Physics* **112**, 5499-5510, doi:doi:http://dx.doi.org/10.1063/1.481116 (2000).
18 Jorgensen, W. L. & Swenson, C. J. Optimized intermolecular potential functions for amides and peptides. Structure and properties of liquid amides. *Journal of the American Chemical Society* **107**, 569-578, doi:10.1021/ja00289a008 (1985).
19 Core Team, R. *R: A language and environment for statistical computing.*, (R Foundation for Statistical Computing, 2014).
20 Baty, F. *et al.* A Toolbox for Nonlinear Regression in R: The Package nlstools. *2015* **66**, 21, doi:10.18637/jss.v066.i05 (2015).
21 Efron, B. & Tibshirani, R. Bootstrap Methods for Standard Errors, Confidence Intervals, and Other Measures of Statistical Accuracy. *Statist. Sci.* **1**, 54-75, doi:10.1214/ss/1177013815 (1986).
